# Funds for Treatment of Hospitalized Patients: Evidence from Bangladesh

**Published:** 2014-09

**Authors:** Farhana Begum, Shahinul Alam, Akmal Hossain

**Affiliations:** ^1^Directorate of Secondary and Higher Secondary Education, Dhaka, Bangladesh; ^2^Department of Hepatology, Bangabandhu Sheikh Mujib Medical University, Shahbag, Dhaka 1000, Bangladesh; ^3^Accounts and Finance, Akij Foot Wear Ltd., Dhaka, Bangladesh

**Keywords:** Developing nations, Healthcare expenditure, Health service, Hospitals, Bangladesh

## Abstract

This study was designed to explore sources of funds for health expenditure of patients if they are hospitalized. We have included 379 patients of 3 private and 7 public hospitals to estimate total expenditure. Of them, 229 (60.4%) were from public and 150 (39.6%) from private hospitals. Mean expenditure was Tk 60,613.3 and 8,262.7, and duration of hospital stay was 10.7 and 11.8 days in private and public hospitals respectively. More than half (55%) of the patients from middle class were treated in private hospitals. Of them, 278 (74.0%) were funded by themselves, 48 (12.8%) by loan with interest rate of 100% to 180%, 23 (6.1%) by loan without interest, 17 (4.5%) by losing their fixed asset, and 4 (1.1%) by begging in the street. Most of the patients did bear expenditure by themselves, followed by loan with high interest rate. ‘Distress’ selling of property was also a source. Middle-class patients could be comfortable with expenditure if they were in public hospitals.

## INTRODUCTION

Health is a basic requirement for improving the quality of life. Economic and social development of a nation mostly depends on the state of health. The health sector occupies an enormously important position to ensure sustainable overall socio-economic advancement in developing countries. In Bangladesh, the Government has begun to integrate the health sector strategically into its poverty reduction plans because an unhealthy nation is meant to continue a vicious cycle of poverty.

Bangladesh is among the least-developed countries with a per-capita income of US$ 621. The health system is not yet well-developed. In developing countries, governments often subsidize services at public healthcare facilities and provide services free of charge to users. However, evidence suggests that users still incur a large expenditure consuming the ‘free’ services for such things that are supposedly provided without charge. Studies have shown that patients incurred substantial out-of-pocket expenditure for medicine, food, and travel for the use of ‘free’ public health facilities (1-3). An overwhelming majority of people in Bangladesh live below the poverty threshold, limiting their access to critical healthcare and other basic needs. Episodes of illness and ill-health may result in substantial medical expenses and trigger impoverishment of households. Cost of healthcare services may deter or delay patients, especially the poor, from seeking appropriate care. Affordability or perceived costs of care are significant factors influencing healthcare behaviours, such as choice of the provider and time of care.

In recent years, the household difficulty in payment for healthcare expenses can result in the ‘distress sale’ of property, delay or abandonment of treatment, and sacrifice expenditure on food and education (3). Other studies have found that introducing or increasing user fees negatively affects the utilization of public health facilities (4-7). Three previous studies have explored issues relating to patients’ expenditure in Bangladesh (3,8,9). World Bank and other donors have been advising developing countries to ensure that limited resources not only have an optimal impact on the people's health at affordable cost but also that health services are client-oriented (10-13). Nahar *et al.* enumerated the patients’ expenditure and affordability of free maternity services for normal delivery and caesarean section (3). Killingsworth *et al*. explored the linkage between official and unofficial fees in public health facilities and concluded that these fees had income and equity effects (14). Stanton *et al.* reviewed literature on user fees and pointed out the need to further investigate the factors and practices causing patients’ expenses before institutional implementation of user fees (14,15).

Recent studies have classified healthcare payments above 10% of income as ‘catastrophic’ for households, assuming that above this threshold, payments are likely to cause cuts to food consumption, debt, and impoverishment. We have designed this study to explore how the people arrange funds for health expenditure if they are hospitalized.

## MATERIALS AND METHODS

### Type and duration of the study

This is a cross-sectional study. The study was done during the period of July 2010 to June 2011.

### Sampling method

The population of the research was defined as Bangladeshis who have been inpatients in public and private hospitals of different cities. The focus dominated on hospitals in Dhaka; this deemed appropriate as Dhaka has the greatest number of hospitals of varying qualities that meet a diverse set of patient needs. Two separate lists of public and private hospitals of Bangladesh were obtained from the Ministry of Health and Family Welfare. From the former list, 7 hospitals, including Dhaka Medical College Hospital, Mitford Hospital, Sher-E-Bangla Medical College Hospital, Jhalokati Sadar Hospital, Babuganj, Razapur and Raipur Upazilla Health Complexes were chosen purposively. Two hospitals in Dhaka have reputation to handle patients from all classes and with various health problems. Dhaka Medical College Hospital is the top and the first public medical college hospital of Bangladesh. Mitford Hospital is also one of the oldest hospitals in Bangladesh. These two are the leading hospitals with the highest patient turnover in the country. Both the hospitals have the longest experiences of patient management in the capital city. The other public hospital selected was Sher-E-Bangla Medical College Hospital which represents one of the large medical college hospitals outside Dhaka, having huge patient-load. Other public hospitals represent the primary and secondary-level public hospitals (Table 1).

**Table 1.** Name of the hospitals and number of patients

Name of hospital	Number of patients
Dhaka Medical College Hospital	50
Mitford Hospital	50
Sher-E-Bangla Medical College Hospital	50
Bangladesh Medical College Hospital	50
Central Hospital	50
Ibn Sina Hospital	50
Jhalokati Sadar Hospital	25
Babuganj Upazilla Health Complex	20
Raipur Upazilla Health Complex	20
Razapur Upazilla Health Complex	14
Total	379

In addition, three hospitals were also purposively chosen from the list of private hospitals. These include Ibn Sina Hospital, Bangladesh Medical College Hospital, and Central Hospital. Bangladesh Medical College Hospital is one of the pinnacle and the first private medical college hospitals in Bangladesh, providing services at an affordable cost. Ibn Sina is also an old hospital with moderate cost. Both are general teaching as well as referral hospitals, with sophisticated technology and skilled manpower. Central Hospital is also serving different classes of patients for a fairly long time. These three hospitals are representatives of private hospitals of the country because lower- and middle-class and rich people can consume the services of these hospitals. There are a few other highly expensive hospitals in the country but these are not accessible to the poor and lower middle-class people; so, these cannot represent services to the general population. To ensure representation, sample-sizes of 50 were selected from every public and private hospital of Dhaka—25 from Jhalokati and 20 from every upazilla health complex but only 14 patients from Razapur health complex agreed to participate in the study. So, the total sample-size was 379. Data were collected only from those respondents who had been admitted as inpatients. Half of the patients were selected from medical wards and the other half from surgical wards. The list of patients ready to be released on a particular date was obtained from the respective ward-in-charge of the hospitals. Using simple random-sampling technique, patients were selected from this list. If one patient was selected from the medical ward, the subsequent patient was selected from the surgical ward. If any patient was unwilling to participate in the study, the successive patient from the same ward was selected.

### Questionnaire design

A preliminary questionnaire was first developed in English, then translated into Bangla. The questionnaire was pretested several times to arrive at appropriate wordings, format, length, and sequencing of the questions (16). Pretest feedback was used in refining the questionnaire until it was ready for data collection.

### Data analysis

The data were analyzed by using Statistical Package for Social Science (version 16) (SPSS Inc., Chicago, IL, USA). Results were expressed as mean±standard deviation (SD) and standard error of mean (SEM). Data were tested for normal distribution. Continuous variables were compared using Independent *t*-test, categorical data were compared by chi-square test, and p<0.05 was considered statistically significant. The summarized information was presented in the form of tables.

## RESULTS

### Demographic characteristics of the study population

We have included a total of 379 patients in our study; male:female ratio was 1:0.84, most of whom were middle-aged, and literacy rate was very low; 167 (44.1%) had primary education only (Table 2). Out of 379 patients, 229 (60.4%) had been admitted to public hospitals, and the rest 150 (39.6%) had been admitted to private hospitals. Of this study population, 193 (50.9%) were managed medically, and 186 (49.1%) required surgery. After treatment, 240 (63.04%) were cured or improved, 97 (25.5%) were as before, and 42 (11.2%) deteriorated from previous condition or died of different diseases.

Occupation of the study population was service: 66 (17.4%), study: 37 (9.8%), business: 36 (9.5%), farming: 33(8.7%), house work 130 (34.3%), and other professions: 77 (20.3%). Most 141 (87.0%) of the poor people were served by the public hospitals, and the affluent 25 (89.2%) were treated in private hospitals. Patients with middle-class socioeconomic status were served by both private [104 (55.0%)] and public [85 (45.0%)] hospitals but mostly by private hospitals. Mean hospital stay was 11.4 days with the range of 1 to 240 day(s). There was no significant difference in duration of hospital stay between private and public hospitals. Hospital stay for surgery was much more than that of those who were medically managed (mean 13.7 and 9.1 days and p<0.005).

### Expenditure for treatment

*Total expenditure*: The total expenditure included the expenditure for treatment, e.g. cost of investigation, medicine, transport, baksheesh (tips), surgery, seat rent, service charge, and any other cost relating to the treatment of the patient. Of the 379 respondents, 6 refused to mention their expenditure to the investigator. Expenditure was far more in private hospitals (Table 2). Total expenditure ranged from Tk 100 to 800,000. Mean expenditure in private hospitals was Tk 60,613.3 and in public hospitals, it was Tk 8,262.7. Cost was significantly higher (p<0.02) if operative treatment was required compared to those treated conservatively; mean±SD (Tk 366,730.0±76,019.4) and (Tk 22,446.1±44,490.6) respectively.

**Table 2.** Demographic characteristics of the study population

Variable	Total	Public hospital	Private hospital
Number	379	229	150
Age (mean±SD) in years	42.4±20.1	39.1±19.2	47.5±20.5
Sex (Male:Female)	206:173	133:96	73:77
Educational status N (%)	
Non-literate	82 (21.6)	56 (24.5)	26 (17.3)
Primary	167 (44.1)	56 (24.5)	26 (17.3)
Secondary	48 (12.7)	113 (49.3)	54 (36.0)
Higher secondary	45 (11.9)	27 (11.8)	21 (14.0)
Higher	37 (9.8)	25 (10.9) 8 (3.5)	20 (13.3) 29 (19.3)
Socioeconomic condition	
Poor	162 (42.7)	141 (61.6)	21 (14.0)
Middle	189 (49.9)	85 (37.1)	104 (69.3)
Rich	28 (7.4)	3 (1.3)	25 (16.7)
Expenditure of treatment in Taka (Mean±SD)	29,036.7±61,996.3	8,262.7±12982.8	60,613.3±88,340.3
Duration of hospital stay in days (Mean±SD)	11.4±15.8	11.8±18.5	10.7±10.4

*Extra payment (tips)*: The clients of the hospitals had to pay extra amount to get the service, which was due for them. It was 161 (70.3%) in public hospitals and 89 (59.3%) in private hospitals. These tips were paid to ayas, ward boys, cleaners, and other lower-class employees.

### Source of fund for expenditure

Most of the patients had to arrange their expenditure by themselves or by the family members (278, 74.1%) out of 376 patients. Of them, source of 252 (67.0%) were themselves or family members only (Table 3). Rest 26 of 278 paid the bill by themselves and family members and from other sources. They had borne the expenditure from current asset, savings, provident fund, fixed deposit, loosing capital asset, and diverting the essential family budget from other heads. None of them had previous budget for this expenditure.

In total, 82 (22.0%) patients raised the fund by getting loan from different sources. Twenty-three (6.1%) of the respondents were financed by loan from persons other than their family members/relatives without interest, and 48 (12.8%) arranged loan from unauthorized persons or organizations. These unauthorized persons are local *Matabbars* or influential persons or distant relatives. They financed these patients with high interest rate, ranging from 100% to 180% per annum. Thirty-two (8.6%) patients had borne the expenditure by donation; of them, 15 (4.0%) received donations from non-government organizations (NGOs) and 17 (4.5%) from unrelated persons. In this study, 4 persons raised the fund by begging in the street or in the localized area. Seventeen (4.5%) of the study population had managed their fund by loosing their fixed asset. They had to sell their land, jewelry, livestock, and other household properties. Three patients did not disclose their source of fund.

## DISCUSSION

Bangladesh is among the least-developed countries with essentially developed beautiful infrastructure in health system. The annual health budget is 6% to 8% of total budget, which is very insufficient to serve its huge population (8). So, the people of this country have to bear the expenditure of their treatment. In addition, people have to pay extra amount for service which is due for them free of charge (3,14). This study is the largest one ever done in Bangladesh on source of fund of the expenditure for treatment.

In this series, we have included 379 patients of primary-, secondary- and tertiary-level hospitals. In previous studies, only tertiary-level hospitals were included. Here, 3 upazilla health complexes have been included, which were the representatives of primary-care hospitals of Bangladesh. These kinds of hospitals were never included in the previous studies. We have tried to make representative sampling from medical and surgical management because, without this, scenarios of expenditure will not be accurate.

**Table 3.** Source of fund for treatment

Source of fund	Frequency	Valid percentage
Self-finance	252	67.0
NGO	15	4.0
Loan from relatives	17	4.5
Donation	14	3.7
Loosing assets	8	2.1
Loan from unauthorized persons	31	8.2
Loan from unauthorized persons + loosing assets	3	0.8
Loan from relatives + loosing assets	4	1.1
Self-finance + loan from relatives	13	3.5
Loan from relatives + loan from unauthorized persons	2	0.5
Self-finance + loan from unauthorized persons	10	2.7
Self-finance + donation	1	0.3
Donation + loan from unauthorized persons	2	0.5
Self-finance + loosing assets	2	0.5
Others	2	0.5

Male:female ratio was 206:173 in this series. This lower proportion of female may be because family members were possibly less interested in treating females. The existing bed distribution in all hospitals of Bangladesh is more for male, which also justifies this ratio in our study. Maximum participants in the study had primary education, and higher educated persons were the lowest in number that had the services of the private hospitals. Half (49.9%) of our respondents were of middle class. Affluent people had chosen the private hospitals for their treatment. These represent the prevailing conditions of Bangladesh. Although the poor people are more in the community, they do not bother to come to the hospitals for their treatment because of unbearable expenditure. Previous studies also explored that increased user fee results in delay or abandonment of treatment of poor people (3-6). More than a half of 104 (55%) middle-class patients were admitted to private hospitals. They could bear the expenditure comfortably if they were in public hospitals instead of private hospitals.

Expenditure for treatment was Tk 100 to 800,000. It was dependent on type of hospital, type of treatment, and duration of hospital stay. Mean expenditure in public hospitals was Tk 8,262.70. It could be lower if they could get the service which was free for them. One hundred and sixty-one (70.3%) of the patients had to pay tips to have the service in public hospitals, which was due for them. Tips were paid to ayas, ward boys, and cleaners. This is in accordance with studies in Bangladesh done by Nahar, Killingsworth, Stanton, and Andaleeb (3,14,15,17). Surprisingly, tips had to be paid by the patients who were treated in private hospitals also.

Most of our respondents (74.0%) had borne the expenditure by themselves, 82 (22.0%) had to take loan for their expenditure, 48 (12.8%) from unauthorized persons. These unauthorized money-lenders are the local influential persons. The interest rate was so high (100% to 180%) that the respondent could never be able to refund it. Part of this scenario was previously been described in another study (3). Four people arranged their funds by begging in the street. Begging in the street for treatment is not uncommon in Bangladesh. Seventeen (4.5%) had to make ‘distress’ sale of their land, livestock, jewelry, and other household assets for health expenditure. This number is lower in our series than that in previous studies (3,14). We have included patients of private hospitals, who were relatively affluent. This is the reason of lower rate of ‘distress’ sale of properties found in our study.

### Conclusions

Most of the patients had borne their expenditure by themselves and family members. A large number of them managed the fund by loosing assets, and ‘distress’ selling was also common. If middle-class patients could be diverted to public hospitals from private ones, the expenditure could be bearable for them. Unauthorized money-lenders invested their money to poor and distressed people, with high interest rates. Tips are very common in the settings of hospital. Good governance in public hospitals may reduce the expenditure. The professionals should take care of the poor patients who are losing their properties during the treatment process. This was a cross-sectional study; we recommend a large-scale study to explore the real situation prevailing in our country.
